# Metabolomic and immunophenotypic signatures in cerebral amyloid angiopathy: a pilot study

**DOI:** 10.1038/s41598-025-31107-w

**Published:** 2025-12-05

**Authors:** Thanos Tsaktanis, Arne Gessner, Steffen Pfeuffer, Daniel Farrenkopf, Ulrike J. Naumann, Kilian Fröhlich, Jochen A. Sembill, Martin F. Fromm, Stefan Schwab, Veit Rothhammer, Joji B. Kuramatsu, Anne Mrochen

**Affiliations:** 1 Department of Neurology, Universitätsklinikum Erlangen, Friedrich-Alexander-Universität Erlangen-Nürnberg (FAU), Erlangen, Germany; 2 Institute of Experimental and Clinical Pharmacology and Toxicology, Friedrich-Alexander-Universität Erlangen-Nürnberg, Erlangen, Germany; 3 Department of Neurology, University Hospital Giessen and Marburg, Justus-Liebig-University Giessen, Giessen, Germany; 4 Department of Neurology, RoMed Klinikum Rosenheim, Rosenheim, Germany

**Keywords:** Cerebral amyloid angiopathy, Metabolomics, Immunphenotyping, Caffeine pathway, Cellular immunity, Stroke, Stroke, Metabolomics, Diagnostic markers

## Abstract

Cerebral amyloid angiopathy (CAA) is a common yet underdiagnosed disease of the small brain vessels, resulting in acute vascular events as well as subcortical neurodegeneration. Currently, it is identified only at advanced stages due to the limitations of non-invasive diagnostic tools. Our pilot study evaluates metabolic and immune alterations in stroke patients with imaging-confirmed CAA. This prospective cohort study included stroke patients admitted to the University Hospital Erlangen with CAA diagnosis based on MRI findings. Metabolomic analysis of cerebrospinal fluid and serum samples was performed using liquid chromatography/mass spectrometry, focusing on caffeine metabolism and amino acid pathways. Immunophenotyping of leukocytes was conducted via flow cytometry. The study included 22 stroke patients, of whom 10 had an MRI-based diagnosis of CAA. Metabolomic analysis revealed consistently lower levels of caffeine-related metabolites in CAA patients with significant differences in 5-acetylamino-6-amino-3-methyluracil, paraxanthine, theobromine, 3,7-dimethyluric acid, 3-methylxanthine and 1-methylxanthine. Amino acid levels showed no significant differences. Immunophenotyping revealed a reduction in CD4^+^ T cell subsets, including effector and central memory T cells, alongside an increase in cytotoxic NK cells in CAA patients. These results suggest that specific metabolic and immune signatures could contribute to the development of diagnostic tools for the detection of CAA.

**Supplementary Information:**

The online version contains supplementary material available at 10.1038/s41598-025-31107-w.

## Introduction

Cerebral amyloid angiopathy (CAA) is a disease affecting the small blood vessels of the brain and affects approximately 50% of individuals over the age of 70 and the vast majority of patients over the age of 90^1^. With the ongoing demographic shift, its prevalence is expected to rise significantly. This is highly relevant, as CAA is recognized as one of the leading causes of lobar intracerebral hemorrhages, lacunar ischemic strokes, and cognitive impairment^2^.

Despite its significance, CAA remains underdiagnosed. A major challenge in diagnosis is the difficulty of establishing a definitive in vivo-diagnosis without invasive tissue sampling^3^. While imaging techniques can detect CAA-specific vascular changes according to the modified Boston criteria, these changes typically become evident only at advanced stages when irreversible brain damage has already occured^3,4^. However, early (asymptomatic) diagnosis is essential for risk stratification, monitoring disease progression, and guiding treatment decisions. Validating biomarkers in imaging-confirmed cases of CAA is a critical first step toward enhancing the sensitivity and specificity of early-stage detection.

Untargeted metabolomics and the development of a disease-specific metabolomic profile have emerged as a promising approach for identifying biomarkers^5–10^. This method enables the detection of metabolites- such as caffeine and amino acids – that have already been evaluated as early diagnostic markers in other neurodegenerative diseases^11,12^. Additionally, a detailed characterization of immune cell populations and their activation states is essential, given the growing evidence linking neuroinflammation to CAA pathogenesis^10^.

To address this issue, our study aims to identify metabolomic and immunological signatures for the detection of CAA. Specifically, we compare stroke patients with imaging-confirmed CAA to those without CAA, as defined by the modified Boston criteria, using (i) flow cytometry to immunophenotype peripheral blood samples and (ii) untargeted metabolomics on cerebrospinal fluid (CSF) and serum samples.

## Results

### Study population

A total of 22 stroke patients were screened, of whom two were excluded due to the absence of MRI.

For the metabolomic analysis, data from 10 patients with CAA (median age 77 years, IQR [74–82], 60% female) and 10 control patients (median age 77 years, IQR [74–82], 40% female) were included.

For immunophenotyping, three additional patients were excluded due to low cell counts, resulting in data from 9 CAA patients (median age 78 years, IQR [74–82]; 56% female) and 8 control patients (median age 76 years, IQR [74–81]; 38% female).

All included patients had an admission diagnosis of ischemic stroke, with approximately 80% classified as transient ischemic attacks and 20% as ischemic strokes; in one patient, concomitant hemorrhagic stroke was also identified. For baseline characteristics, including clinical and imaging parameters see also Supplementary Tables 1 and 2.

### Selection of metabolic pathways for analysis

Through untargeted metabolomics profiling, we identified a diverse range of metabolites. For our analysis, we specifically selected two metabolic pathways: (i) Caffeine metabolism, due to its neuroprotective properties, including antioxidant activity and modulation of neuroinflammatory pathways, which have been implicated in neurodegenerative diseases such as Parkinson’s disease^11,13,14^ and (ii) Amino acid metabolism, as disruptions in this pathway—particularly in branched-chain amino acids—have been associated with neurodegenerative diseases^15–17^.

### Caffeine metabolism metabolites reduced in CAA patients

**Fig. 1:**
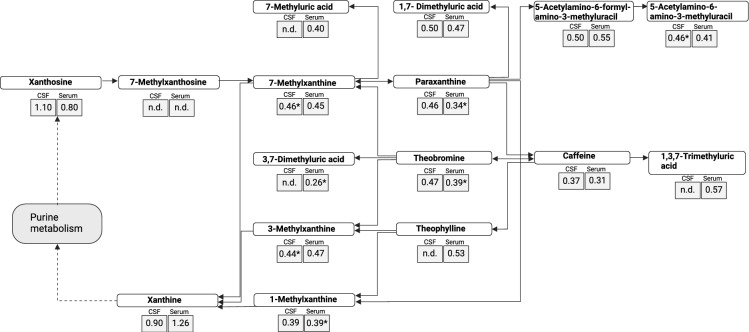
Metabolomic analysis of the caffeine pathway. Metabolites of the caffeine pathway in cerebrospinal fluid (CSF) and serum samples from patients with cerebral amyloid angiopathy (CAA) compared to controls. The data is presented as ratio of CAA patients to control subjects based on the normalized peak area detected for each metabolite^9^. Metabolites that showed significant differences between CAA patients and controls (*p < 0.05) are marked with an asterisk. “n.d.” indicates non-detectable levels.

Each caffeine metabolism metabolite is presented as a ratio between CAA patients and controls based on the normalized peak area, measured in both serum and CSF (Fig. 1). The ratios of caffeine metabolism-related metabolites in CSF and serum were consistently below 1, indicating lower concentrations of all metabolites in CAA patients compared to control patients. The following metabolites showed significant reductions (p < 0.05): 5-acetylamino-6-amino-3-methluracil (CSF), paraxanthine (serum), theobromine (serum), 3,7-dimethyluric acid (serum), 3-methylxanthine (CSF), 1-methylxanthine (serum), suggesting a relevance of caffeine metabolism and a potential dysregulation of this pathway.

### Amino acids profiles unchanged in CAA patients

**Fig. 2:**
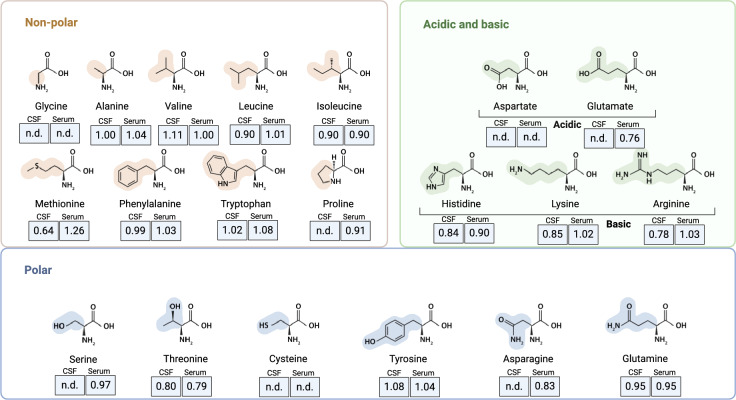
Metabolomic analysis of amino acids. Amino acids in cerebrospinal fluid (CSF) and serum samples from patients with cerebral amyloid angiopathy (CAA) compared to controls. The data is presented as the ratio (CAA/controls) of the analyzed metabolites. No significant differences were observed for all amino acids between CAA and controls. “n.d.” indicates non-detectable levels.

Each amino acid is presented as a ratio between CAA patients and controls based on the normalized peak area, measured in both serum and CSF (Fig. 2). We found no significant difference between CAA and controls (p > 0.05 for all comparisons), suggesting no significant metabolic alterations in this context.

### Immune cell populations in CAA patients

**Fig. 3:**
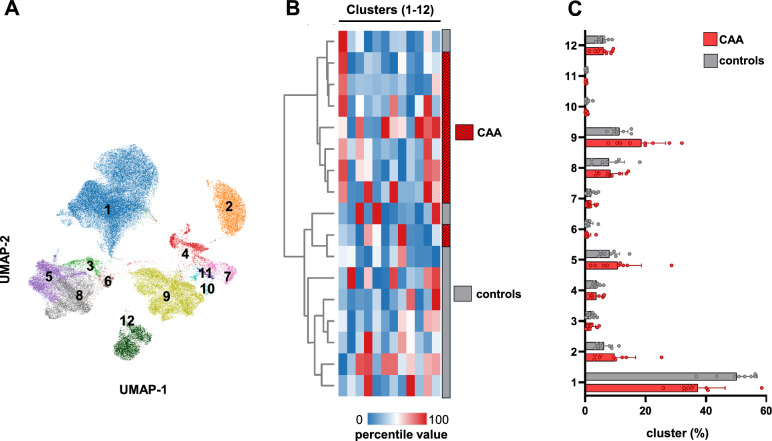
Multiparametric clustering reveals distinct immune cell profiles in blood samples of CAA patients compared to control patients. (**A**) Unsupervised UMAP (Uniform Manifold Approximation and Projection) projection of CAA (n = 9) and control patients (n = 8) followed by FlowSOM clustering (k = 12). (**B**) Hierarchical clustering by Euclidean distance. Clustering was performed based on the relative abundance of FlowSOM clusters (k = 12) per sample. Compared were control patients (n = 8) and CAA patients (n = 9). (**C**) Relative abundance of FlowSOM clusters (k = 12) in control patients (n = 8) and CAA patients (n = 9).

Advanced immunophenotyping of peripheral blood samples from 9 CAA patients and 8 control patients was performed to investigate potential alterations in immune cell subsets. Initially, a comprehensive and unbiased analysis was conducted to systematically assess changes in adaptive and innate immune responses via unsupervised dimensionality reduction (Fig. 3A). Following this, hierarchical clustering using Euclidean distance—based on the relative abundance of FlowSOM-identified clusters—identified 12 immune cell clusters (Fig. 3B). These clusters were characterized by the expression of surface markers CD16, HLA-DR, CD45RA, CD45, CD3, CD8, CD56, CCR7, CD27, CD14, CD19 and IgD (Supplementary Fig. 1A).

The analysis revealed significant alterations in specific immune cell clusters. Cluster 1 was significantly reduced in CAA patients, while Cluster 9 was elevated compared to controls (Fig. 3C). Based on surface marker expression (Supplementary Fig. 1A), Cluster 1 was identified as CD4^+^ T cells, while Cluster 9 corresponded to natural killer (NK) cells. Given their critical role in immune regulation, these clusters were analyzed in greater detail.

**Fig. 4:**
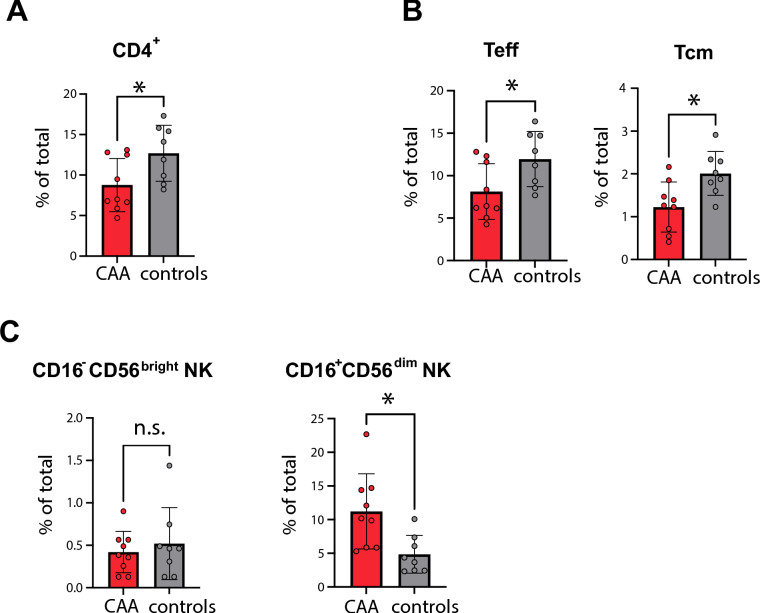
CD4 + subsets and cytotoxic CD16 + NK cells are differentially regulated in CAA patients compared to control patients. Frequencies (% of total) of (**A**) CD4^+^ T helper cells (**B**) Teff; CD4^+^ effector T cells (CD3^+^ CD4^+^CD127^+^CD56^–^CD8^–^CD25^+^), TCM; central memory CD4^+^ T cells (CD3^+^CD4^+^CCR7^+^CD45RA^–^) (**C**) CD56^bright^CD16^-^ regulatory and CD56^dim^CD16^+^ cytotoxic NK cells. Unpaired two-tailed *t*-test with Welch’s correction; *p < 0.05 are marked with an asterisk. “ns” indicates non significant.

In CAA patients, CD4^+^ T cells showed a marked reduction (Fig. 4A), particularly within the effector T cell (Teff) population and the central memory CD4^+^ T cell (TCM) subset Fig. 4B). In contrast, frequencies of other adaptive immune cell subsets, including CD8^+^ T cells, Tregs (CD3^+^CD4^+^CD25^+^CD56^–^CD8^–^CD127^–^), CD4^+^ Tn (naïve CD4^+^ T cells; CD45RA^+^CCR7^+^), CD4^+^ TEM (effector memory CD4^+^ T cells; CCR7^–^CD45RA^–^), and CD4^+^ TEMRA (effector memory CD4^+^ T cells re-expressing CD45RA; CD45RA^+^CCR7^–^), remained unchanged between CAA patients and controls (Supplementary Figs. 2A,B).

Innate immune responses were also examined, revealing a significant increase in CD56^dim^CD16^+^ NK cells in CAA patients, a phenotype associated with heightened cytotoxic functionality. In contrast, the frequency of CD56^bright^CD16^–^ NK cells, which are known for their regulatory functions, remained unchanged (Fig. 4C).

To further explore immune cell population dynamics, B cell subsets, dendritic cells (DCs), and monocytes were analyzed. No significant differences were observed between CAA and control patients in B cell subsets, including naïve B cells (IgD^+^CD27^–^), memory B cells (CD27^+^IgD^–^), IgD^+^ memory B cells (CD27^+^IgD^+^), and double-negative B cells (CD27^–^IgD^–^), as well as DCs (HLA-DR^+^CD11c^+^) and monocyte populations, comprising classical (CD14^+^CD16^–^), intermediate (CD14^+^CD16^+^), and non-classical monocytes (CD16^+^CD14^–^) (Supplementary Figs. 2C–E).

In summary, immune profiling in CAA patients revealed a significant reduction in CD4^+^ T cells, particularly within the effector and central memory subsets, while other adaptive immune cell populations remained unchanged, alongside a marked increase in cytotoxic NK cells without alterations in regulatory NK cell subsets, suggesting a shift from adaptive immunity toward enhanced innate immune activation.

## Discussion

In this study, we aimed to identify a specific signature of CAA in blood and cerebrospinal fluid of patients with imaging confirmed CAA by integrating untargeted metabolomics and immunophenotyping by flow cytometry. Our analysis revealed several key findings. First, caffeine and its metabolites were observed at consistently lower levels in both CSF and serum of CAA patients compared to controls, suggesting a potential alteration in caffeine metabolism specific to this patient group. Second, no significant differences were found in the levels of amino acids, including leucine, between CAA patients and controls. Third, immune profiling revealed a significant decrease in CD4^+^ T cells, including effector and central memory subsets, coupled with an increase in cytotoxic NK cells in CAA patients compared to control patients.

Our first finding revealed consistently lower levels of caffeine and its metabolites in the CSF and serum of CAA patients compared to controls. This indicates that caffeine could serve as a crucial component of a metabolomic signature for the detection of CAA. However, it is important to note that we did not adjust for caffeine consumption between the groups, meaning that we cannot exclude the possibility that the observed differences in caffeine and its metabolites may reflect variations in consumption habits. Nevertheless, it is noteworthy that in Parkinson’s disease, coffee consumption patterns have been discussed as a potential factor influencing the risk of disease development during the prodromal phase^11,18,19^. This raises the possibility that similar caffeine-related behaviors may also play a role in other diseases, such as CAA. One potential explanation is that caffeine and its metabolites, particularly paraxanthine, have been shown to exhibit neuroprotective properties through mechanisms such as antioxidant activity and the modulation of neuroinflammatory pathways^11,13,20^. In Parkinson’s disease, caffeine intake has been associated with a reduced risk of disease onset and slower progression, possibly due to its effects on adenosine receptors, which are involved in regulating neuroinflammation and neuronal excitability^13,19,21^. Caffeine may also confer neuroprotection in Alzheimer’s disease by mitigating β-amyloid-induced neuronal damage through adenosine A₂A receptor inhibition^22^. Similar mechanisms may operate in CAA, where amyloid-driven vascular inflammation and endothelial dysfunction underlie neurovascular injury.

Importantly, our study revealed that the ratios of caffeine metabolites were remarkably similar between CSF and serum, indicating that peripheral measurements could reliably reflect central caffeine metabolism in CAA patients.

Our second finding revealed no significant differences in the levels of amino acids between CAA patients and controls. This finding contrasts with some other neurodegenerative diseases, where disruptions in branched-chain amino acid metabolism have been observed^17^. Notably, a genetic predisposition to raised plasma isoleucine is positively associated with Alzheimers Disease^15^. Furthermore, the acetylated variant of leucine, N-acetyl-L-leucine, has recently gained attention for its potential role in modulating energy metabolism, with therapeutic promise attributed to its ability to enhance neuronal function and provide neuroprotection^16^. Additionally, alterations in other amino acid pathways, such as tryptophan metabolism, may contribute to adaptive anti-inflammatory responses, potentially driving inflammaging and underlying the pathophysiology of age-related diseases^23,24^. In contrast, in our study, no significant differences in amino acid levels were found between CAA patients and controls, which may be attributed to small sample sizes, preventing definitive conclusions about its contribution to a metabolomic signature for CAA patients.

Our third finding revealed a significant reduction in CD4^+^ T cells (including effector, regulatory, and central memory subsets) in CAA patients compared to controls. Concurrently, CAA patients exhibited an expansion of cytotoxic NK cells, particularly within the CD56^dim^CD16^+^ population, indicating a shift toward enhanced innate cytotoxic responses.

NK cells are key innate immune effectors, mainly known for their role in fighting infections and tumors^25,26^. Their involvement in neurodegenerative diseases like CAA is less clear. Amyloid-induced vascular damage in CAA may lead to endothelial dysfunction and blood–brain barrier disruption, facilitating immune cell infiltration^27–29^. In particular, cytotoxic CD56^dim^CD16^+^ NK cells may interact with T and B cells, promoting neuroinflammation and potentially autoimmune responses^30^. While NK cells can remove damaged cells, their activation in inflammatory settings may worsen blood–brain barrier damage and contribute to disease progression in CAA^26,31^. Collectively, these findings suggest an altered immune response in CAA, characterized by diminished helper T cell subsets and an increase in innate cytotoxic activity. This shift suggests a potential compensatory mechanism to address the deficits in adaptive immunity^32^. Notably, monocyte, B cell, and dendritic cell populations exhibited no alteration, indicating that these components of the immune system remain unaffected in CAA. Together, these findings highlight alterations in both adaptive and innate immune responses in CAA, with a distinct shift toward heightened cytotoxic activity within the innate immune system. However, the role of the immune system in CAA remains not entirely clear^10^. While the immune system plays a well-defined role in the rare inflammatory forms of CAA, such as CAA-related inflammation and Aβ-related angiitis, immune system involvement is also evident in the more common forms of CAA^33^. The immune system can also contribute to the pathophysiology of CAA, exacerbating vascular injury, compromising the blood–brain barrier, and promoting inflammation, which can accelerate the progression of CAA. These findings further emphasize the complex interplay of immune responses in CAA as observed in the changes to adaptive and innate immune profiles in our study.

While our study provides novel insights, several limitations should be acknowledged. First, this was a pilot study with a small sample size, particularly in the immunophenotyping cohort, which limits the generalizability of our findings. The exclusion of some samples due to low cell counts further reduced the statistical power of the immunophenotyping analysis, and the low cell counts may introduce confounding, as our analyses reflect only a brief snapshot of the current metabolic and immunological state. To ensure a more comprehensive clinical characterization, future studies should include cognitive assessments alongside biochemical profiling. Overall, the reduced statistical power underscores the need for validation in larger, independent cohorts to confirm the observed metabolic and immune alterations in CAA. Additionally, caffeine intake was not assessed or controlled for, posing a potential confounder. The observed reduction in caffeine metabolites may reflect differences in consumption rather than disease-specific alterations. Future studies should account for caffeine intake to clarify its relevance in CAA pathophysiology. Our metabolomic analyses were cross-sectional, restricting our ability to infer causality or temporal changes in metabolite profiles during disease progression. Longitudinal studies are warranted to determine whether these metabolic alterations precede, accompany, or result from disease processes in CAA.

Additionally, we focused on only a few specific metabolic pathways. A much larger sample size and broader analysis of multiple metabolic pathways will be essential to establish a comprehensive and specific metabolomic signature for CAA patients. We did not apply correction for multiple comparisons, which increases the risk of false-positive results and may lead to overinterpretation of some findings. However, in our case, several significant metabolic features converge within the same pathway, which we interpret as a biologically coherent and consistent effect. Notably, there is also a possibility that some control participants could have undiagnosed CAA, as imaging changes often appear at later stages of the disease. However, this would likely result in false-negative findings.

Nevertheless, as a pilot study, our work provides a foundation for future research, highlighting the need for larger, longitudinal studies to validate these findings and refine metabolomic profiling for early and accurate CAA diagnosis.

In conclusion, our findings highlight alterations in immune and metabolic signatures in CAA patients, emphasizing the interplay between immune dysfunction and metabolic changes in disease pathology. Importantly, these results suggest that specific metabolic and immune signatures could contribute to the development of diagnostic tools for the early detection of CAA. Furthermore, it would be particularly interesting from a future perspective to profile these immune and metabolic signatures across different clinical phenotypes, such as lobar hemorrhage, lacunar ischemia, and cognitive deficits.

## Materials and methods

### Study design and participants

This prospective cohort analysis was conducted on patients admitted to the Department of Neurology at the University Hospital Erlangen between January 2022 and October 2023. The study protocol (Nr. 20_21 Bc) received approval from the Ethics Committee of the University Erlangen-Nuremberg. Inclusion criteria were: (i) diagnosis of a transient ischemic attack (TIA) or ischemic/hemorrhagic stroke, and (ii) an MRI scan performed within a 6-month period either before or after hospital admission. Patients were classified as having CAA patients based on imaging findings and categorized as ‘probable’ according to the modified Boston criteria^3,34^ (Supplementary Table 2). Supplementary cerebrospinal fluid and peripheral blood samples were concurrently collected as part of the diagnostic procedure within the first 0–2 days after hospital admission (Supplementary Table 1).

### Metabolomic analysis

#### Sample collection and processing

CSF and serum samples were collected following stringent protocols to minimize metabolic alterations. Each sample was processed within a standardized timeframe of 30 min post-collection to ensure the integrity of the metabolites. For CSF samples, 600 µl aliquots were prepared and stored at –80 °C immediately until further analysis. Serum samples were subjected to centrifugation at 2750 rcf at 15 °C for 10 min. The supernatant was collected, aliquoted in 600 µl volumes, and stored at –80 °C.

#### Analysis: untargeted metabolomics

Analysis was performed at the Core Unit METAB of the Medical Faculty at FAU Erlangen using liquid chromatography-mass spectrometry (LC–MS), following previously established protocols^8,9^. Data analysis was conducted using Compound Discoverer 3.3 (Thermo Fisher Scientific, Dreieich, Germany)^9^. All peak areas were normalized to signals obtained from quality control (QC) samples. For each of the metabolites presented, the ratio of CAA patients to control subjects is shown based on the normalized peak area detected for each metabolite. A ratio greater than 1 indicates that the metabolite is present at a higher concentration in the patient group compared to the control group.

Analysis focused on features that were annotated with ID levels 1 or 2 as proposed by Schymanski^20^. ID level 1 annotations are supported by MS and chromatography data from reference standards analyzed on the same system and included in an in-house library, ensuring unambiguous identification. ID level 2 annotations show very strong agreement with experimental MS data in HMDB, yet lack confirmation from a reference standard.

This untargeted metabolomic analysis provided an overview of metabolic alterations in cerebrospinal fluid and serum samples from stroke patients with and without imaging-confirmed CAA. Following an exploratory analysis, we selected two key metabolic pathways—specifically the caffeine metabolism pathway and amino acid metabolisms^11–13,15^.

### Flow cytometry analysis of leukocytes

#### Sample collection and processing

Whole blood was collected and processed using ACK (Ammonium-Chloride-Potassium) lysis buffer (ThermoFisher) according to the manufacturer’s protocol to lyse erythrocytes while preserving leukocytes. Following lysis, cell suspensions were cryopreserved in liquid nitrogen under standardized conditions. Cell viability was consistently maintained above 80%, as confirmed by routine quality assessments comparing frozen leukocyte samples to freshly isolated leukocytes throughout the study. This ensured the reliability and reproducibility of subsequent analyses.

#### Analysis: flow cytometric analysis

Flow cytometric analysis was conducted following a standardized protocol^35^. Frozen peripheral leukocytes were thawed, incubated at 37 °C in medium, and centrifuged at 300 g for 10 min^36^. Live/dead staining was performed with LIVE/DEAD Zombie NIR (Biolegend) according to the manufacturer’s instructions. The cells were resuspended in FACS buffer consisting of phosphate-buffered saline (PBS) (PAA, Pasching, Austria) supplemented with 2% fetal calf serum (FCS) (Invitrogen, Darmstadt). Subsequently, cells were stained at 4 °C for 30 min, washed, and then analyzed using Cytek Northern Lights (Cytek Biosciences, Fremont, CA, USA) flow cytometer. Various antibodies were used for staining, including CD235a-PE/Dazzle594 (Biolegend; clone HI264), CD 3 cFluor V420 (Cytek; clone SK7), CD4-cFluor R780 (Cytek; clone SK3), CD8-cFluor B515 (SK1), CD127-cFluor R659 (Cytek; clone eBioRDR5), CD25-cFluor BYG781 (Cytek; clone BC96), CD45RA-cFluor B690 (Cytek, clone HI100), CCR7-cFluor BYG575 (Cytek, clone G043H7), CD56-cFluorR720 (Cytek; clone 5.1H11), CD19 cFluor-BYG710 (Cytek; clone HIB19), CD16 cFluor-R668 (Cytek, clone 3G8), IgD cFluor-BYG667 (Cytek, clone IgD26), CD27-cFluor R840 (Cytek; clone O323), HLA-DR-Super Bright 600 (Biolegend; clone L243), CD14-cFluor V450 (Cytek; clone MEM-15), CD11c-BV711 (Biolegend; clone 3.9) as suggested^35^. A detailed gating strategy is provided in Supplementary Fig. 1B. The immune cell clusters identified in this study are based on well-established surface markers that have been extensively utilized in prior research^36^. These clusters are consistent with those described in the existing literature^35^.

#### Analysis of multiparametric flow cytometry data using the OMIQ platform

Flow Cytometry Standard files were exported and analyzed using the OMIQ platform as previously described^36,37^. Following scaling, samples were down-sampled to 100,000 live cells per group and analyzed through either manual gating or unsupervised methods. Opt-SNE analysis was performed with 560 iterations, a perplexity value of 30, and a theta parameter of 0.5. This was followed by Consensus Metaclustering using FlowSOM, with the number of clusters (k) set to 20.

#### Statistical methods

The log10-transformed area values of individual metabolites for each patient were used to perform an unpaired *t*-test using Compound Discoverer Software 3.3 (Thermo Fisher Scientific). This analysis was conducted to compare the differences between the CAA patient group and the control group.

Statistical analyses were conducted using R version 4.3.3 and GraphPad Prism version 9.5.1. Figures (Figs. 1 and 2) were created using BioRender.com. Besides dimensionality-reduction analysis as described above, relative cell proportion of immune cell subpopulations were compared using an unpaired two-tailed t-test with Welch’s correction.

All statistical tests were two-tailed, and *p*-values less than 0.05 were considered statistically significant. All values were considered exploratory and thus, we refrained from correction for multiple testing.

## Supplementary Information

Supplementary Information.

## Data Availability

The data that support the findings of this study are available from the corresponding author, A.M., upon reasonable request.

## References

[1] Charidimou, A. et al. Emerging concepts in sporadic cerebral amyloid angiopathy. *Brain* **140**, 1829–1850. 10.1093/brain/awx047 (2017). 28334869 10.1093/brain/awx047 PMC6059159

[2] Schreiber, S. et al. Invited review: The spectrum of age-related small vessel diseases: Potential overlap and interactions of amyloid and nonamyloid vasculopathies. *Neuropathol. Appl. Neurobiol.* **46**, 219–239. 10.1111/nan.12576 (2020). 31386773 10.1111/nan.12576

[3] Greenberg, S. M. & Charidimou, A. Diagnosis of cerebral amyloid angiopathy: Evolution of the Boston criteria. *Stroke* **49**, 491–497. 10.1161/strokeaha.117.016990 (2018). 29335334 10.1161/STROKEAHA.117.016990 PMC5892842

[4] Koemans, E. A. et al. Progression of cerebral amyloid angiopathy: A pathophysiological framework. *Lancet Neurol.* **22**, 632–642. 10.1016/s1474-4422(23)00114-x (2023). 37236210 10.1016/S1474-4422(23)00114-X

[5] Buergel, T. et al. Metabolomic profiles predict individual multidisease outcomes. *Nat. Med.* **28**, 2309–2320. 10.1038/s41591-022-01980-3 (2022). 36138150 10.1038/s41591-022-01980-3 PMC9671812

[6] Castellanos-Jankiewicz, A. Improving predictions for risk of common diseases through metabolomics.

[7] Banerjee, G. et al. Clinical considerations in early-onset cerebral amyloid angiopathy. *Brain* **146**, 3991–4014. 10.1093/brain/awad193 (2023). 37280119 10.1093/brain/awad193 PMC10545523

[8] Gessner, A. et al. New biomarkers for renal transporter-mediated drug-drug interactions: Metabolomic effects of cimetidine, probenecid, verapamil, and rifampin in humans. *Clin. Pharmacol. Ther.*10.1002/cpt.3414 (2024). 39148267 10.1002/cpt.3414 PMC11652812

[9] Gessner, A. et al. A metabolomic analysis of sensitivity and specificity of 23 previously proposed biomarkers for renal transporter-mediated drug-drug interactions. *Clin. Pharmacol. Ther.* **114**, 1058–1072. 10.1002/cpt.3017 (2023). 37540045 10.1002/cpt.3017

[10] Munsterman, D. et al. Cerebral amyloid angiopathy and the immune system. *Alzheimers Dement.* **20**, 4999–5008. 10.1002/alz.13826 (2024). 38881491 10.1002/alz.13826 PMC11247707

[11] Fujimaki, M. et al. Serum caffeine and metabolites are reliable biomarkers of early Parkinson disease. *Neurology* **90**, e404–e411. 10.1212/wnl.0000000000004888 (2018). 29298852 10.1212/WNL.0000000000004888 PMC5791797

[12] Socha, E., Koba, M. & Kośliński, P. Amino acid profiling as a method of discovering biomarkers for diagnosis of neurodegenerative diseases. *Amino Acids* **51**, 367–371. 10.1007/s00726-019-02705-6 (2019). 30725224 10.1007/s00726-019-02705-6

[13] Chen, J. F. et al. Neuroprotection by caffeine and A(2A) adenosine receptor inactivation in a model of Parkinson’s disease. *J. Neurosci.* **21**, Rc143. 10.1523/JNEUROSCI.21-10-j0001.2001 (2001). 11319241 10.1523/JNEUROSCI.21-10-j0001.2001 PMC6762498

[14] Xu, K., Xu, Y. H., Chen, J. F. & Schwarzschild, M. A. Neuroprotection by caffeine: Time course and role of its metabolites in the MPTP model of Parkinson’s disease. *Neuroscience* **167**, 475–481. 10.1016/j.neuroscience.2010.02.020 (2010). 20167258 10.1016/j.neuroscience.2010.02.020 PMC2849921

[15] Larsson, S. C. & Markus, H. S. Branched-chain amino acids and Alzheimer’s disease: A Mendelian randomization analysis. *Sci. Rep.* **7**, 13604. 10.1038/s41598-017-12931-1 (2017). 29051501 10.1038/s41598-017-12931-1 PMC5648806

[16] Oertel, W. H. et al. Acetyl-DL-leucine in two individuals with REM sleep behavior disorder improves symptoms, reverses loss of striatal dopamine-transporter binding and stabilizes pathological metabolic brain pattern-case reports. *Nat. Commun.* **15**, 7619. 10.1038/s41467-024-51502-7 (2024). 39223119 10.1038/s41467-024-51502-7 PMC11369233

[17] Yan, Z. et al. Longitudinal metabolomics profiling of serum amino acids in rotenone-induced Parkinson’s mouse model. *Amino Acids* **54**, 111–121. 10.1007/s00726-021-03117-1 (2022). 35028704 10.1007/s00726-021-03117-1

[18] Ross, G. W. et al. Association of coffee and caffeine intake with the risk of Parkinson disease. *JAMA* **283**, 2674–2679. 10.1001/jama.283.20.2674 (2000). 10819950 10.1001/jama.283.20.2674

[19] Zhao, Y. et al. Association of coffee consumption and prediagnostic caffeine metabolites with incident Parkinson disease in a population-based cohort. *Neurology* **102**, e209201. 10.1212/wnl.0000000000209201 (2024). 38513162 10.1212/WNL.0000000000209201 PMC11175631

[20] Pardo, M., Paul, N. E., Collins-Praino, L. E., Salamone, J. D. & Correa, M. The non-selective adenosine antagonist theophylline reverses the effects of dopamine antagonism on tremor, motor activity and effort-based decision-making. *Pharmacol. Biochem. Behav.* **198**, 173035. 10.1016/j.pbb.2020.173035 (2020). 32910928 10.1016/j.pbb.2020.173035

[21] Ascherio, A. et al. Prospective study of caffeine consumption and risk of Parkinson’s disease in men and women. *Ann. Neurol.* **50**, 56–63. 10.1002/ana.1052 (2001). 11456310 10.1002/ana.1052

[22] Dall’Igna, O. P. et al. Caffeine and adenosine A(2a) receptor antagonists prevent beta-amyloid (25–35)-induced cognitive deficits in mice. *Exp. Neurol.* **203**, 241–245. 10.1016/j.expneurol.2006.08.008 (2007). 17007839 10.1016/j.expneurol.2006.08.008

[23] Cervenka, I., Agudelo, L. Z. & Ruas, J. L. Kynurenines: Tryptophan’s metabolites in exercise, inflammation, and mental health. *Science*10.1126/science.aaf9794 (2017). 28751584 10.1126/science.aaf9794

[24] Sorgdrager, F. J. H., Naudé, P. J. W., Kema, I. P., Nollen, E. A. & Deyn, P. P. Tryptophan metabolism in inflammaging: From biomarker to therapeutic target. *Front. Immunol.* **10**, 2565. 10.3389/fimmu.2019.02565 (2019). 31736978 10.3389/fimmu.2019.02565 PMC6833926

[25] Böttcher, J. P. et al. NK cells stimulate recruitment of cDC1 into the tumor microenvironment promoting cancer immune control. *Cell* **172**, 1022-1037.e1014. 10.1016/j.cell.2018.01.004 (2018). 29429633 10.1016/j.cell.2018.01.004 PMC5847168

[26] Chen, S., Zhu, H. & Jounaidi, Y. Comprehensive snapshots of natural killer cells functions, signaling, molecular mechanisms and clinical utilization. *Signal Transduct. Target. Ther.* **9**, 302. 10.1038/s41392-024-02005-w (2024). 39511139 10.1038/s41392-024-02005-w PMC11544004

[27] Switzer, A. R. et al. Longitudinal decrease in blood oxygenation level dependent response in cerebral amyloid angiopathy. *NeuroImage Clin.* **11**, 461–467. 10.1016/j.nicl.2016.02.020 (2016). 27104140 10.1016/j.nicl.2016.02.020 PMC4827726

[28] Shin, H. K. et al. Age-dependent cerebrovascular dysfunction in a transgenic mouse model of cerebral amyloid angiopathy. *Brain* **130**, 2310–2319. 10.1093/brain/awm156 (2007). 17638859 10.1093/brain/awm156

[29] Lepennetier, G. et al. Cytokine and immune cell profiling in the cerebrospinal fluid of patients with neuro-inflammatory diseases. *J Neuroinflamm.* **16**, 219. 10.1186/s12974-019-1601-6 (2019). 10.1186/s12974-019-1601-6 PMC6857241 31727097

[30] Schafflick, D. et al. Integrated single cell analysis of blood and cerebrospinal fluid leukocytes in multiple sclerosis. *Nat. Commun.* **11**, 247. 10.1038/s41467-019-14118-w (2020). 31937773 10.1038/s41467-019-14118-w PMC6959356

[31] Vivier, E., Tomasello, E., Baratin, M., Walzer, T. & Ugolini, S. Functions of natural killer cells. *Nat. Immunol.* **9**, 503–510. 10.1038/ni1582 (2008). 18425107 10.1038/ni1582

[32] Jiang, H. & Jiang, J. Balancing act: the complex role of NK cells in immune regulation. *Front. Immunol.* **14**, 1275028. 10.3389/fimmu.2023.1275028 (2023). 38022497 10.3389/fimmu.2023.1275028 PMC10652757

[33] Chwalisz, B. K. Cerebral amyloid angiopathy and related inflammatory disorders. *J. Neurol. Sci.* **424**, 117425. 10.1016/j.jns.2021.117425 (2021). 33840507 10.1016/j.jns.2021.117425

[34] Charidimou, A. et al. The Boston criteria version 2.0 for cerebral amyloid angiopathy: A multicentre, retrospective, MRI-neuropathology diagnostic accuracy study. *Lancet Neurol.* **21**, 714–725. 10.1016/s1474-4422(22)00208-3 (2022). 35841910 10.1016/S1474-4422(22)00208-3 PMC9389452

[35] Maecker, H. T., McCoy, J. P. & Nussenblatt, R. Standardizing immunophenotyping for the human immunology project. *Nat. Rev. Immunol.* **12**, 191–200. 10.1038/nri3158 (2012). 22343568 10.1038/nri3158 PMC3409649

[36] Tsaktanis, T. et al. Regulation of the programmed cell death protein 1/programmed cell death ligand 1 axis in relapsing-remitting multiple sclerosis. *Brain Commun.* **5**, fcad206. 10.1093/braincomms/fcad206 (2023). 37564830 10.1093/braincomms/fcad206 PMC10411318

[37] Linnerbauer, M. et al. The astrocyte-produced growth factor HB-EGF limits autoimmune CNS pathology. *Nat. Immunol.* **25**, 432–447. 10.1038/s41590-024-01756-6 (2024). 38409259 10.1038/s41590-024-01756-6 PMC10907300

