# Supplementary material for: Metabolomic and immunophenotypic signatures in cerebral amyloid angiopathy: a pilot study
Source: Sci Rep. 2025 Dec 5;15:43262. doi: 10.1038/s41598-025-31107-w (PMC12685982; doi:10.1038/s41598-025-31107-w)
Supplement: Supplementary file 1 — Supplementary Information. [file 41598_2025_31107_MOESM1_ESM.pdf]

	Metabolomics			Immunphenotyping		
**Characteristics**	**control patient (n=10)**	**CAA (n=10)**	**SMD**	**control patient (n=8)**	**CAA (n=9)**	**SMD**
Age, years, median (IQR)	77 (72-79)	77 (74-82)	0.05	76 (74-81)	78 (74-82)	0.08
Sex, female, n (%)	4 (40)	6 (60)	0.41	3 (38)	5 (56)	0.37
Specimen Collection Date (Since Admission in days)	1 (0-1)	1 (0-2)	0.59	1 (0-1)	1 (0-2)	0.70
**Admission diagnosis**						
Intracerebral hemorrhage, n (%)	0 (0.0)	1 (10)	0.47	0 (0.)	1 (11)	0.50
Ischemic stroke, n (%)	2 (20)	2 (20)	0.01	2 (25)	2 (22)	0.07
Transient ischemic attack, n (%)	8 (80)	8 (80)	0.01	6 (75)	6 (66)	0.07
**Prior medical history**						
Arterial hypertension, n (%)	7 (70)	8 (80)	0.23	6 (75)	7 (78)	0.07
Diabetes mellitus, n (%)	5 (50)	6 (60)	0.20	5 (63)	5 (56)	0.14
Coronary artery disease, n (%)	7 (70)	3 (30)	0.01	6 (75)	2 (22)	> 0.5
History of smoking, n (%)	2 (20)	2 (20)	0.01	2 (25)	1 (11)	0.37
History of alcohol use, n (%)	1 (10)	1 (10)	0.01	1 (1%)	1 (11)	0.04
**Prior medication**						
Antiplatelet therapy, n (%)	7 (70)	5 (50)	0.42	5 (63)	5 (56)	0.14
Oral anticoagulants, n (%)	3 (30)	1 (10)	0.51	3 (38)	1 (11)	> 0.5
Statins, n (%)	8 (80)	8 (80)	0.01	7 (88)	7 (78)	0.26

Supplementary Table 1. Characteristics of CAA patients and control patients. Abbreviations: cerebral amyloid angiopathy, CAA; interquartile range, ICR; standardized mean difference, SMD

	Metabolomics		Immunphenotyping	
**Characteristics**	**control patient (n=10)**	**CAA (n=10)**	**control patient (n=8)**	**CAA (n=9)**
Cerebral microbleeds, n (%)	0 (0)	10 (100)	0 (0)	9 (100)
Lobar, n (%)	0 (0)	10 (100)	0 (0)	9 (100)
Cerebellar, n (%)	0 (0)	2 (20)	0 (0)	2 (22)
Superficial siderosis, n (%)	0 (0)	10 (100)	0 (0)	9 (100)
Focal, n (%)	0 (0)	7(70)	0 (0)	7 (78)
Disseminated, n (%)	0(0)	3(30)	0 (0)	2 (22)
Prior cerebral ischemia, n (%)	0 (0)	6 (60)	5 (56)	6 (67)
Fazekas score, median (IQR)	1 (1-2)	1 (1-2)	1 (1-3)	1 (1-2)
Probable CAA*, n (%)	0 (0)	10 (100)	0 (0)	9 (100)

Supplementary Table 2. MRI characteristics of CAA patients and control patients. Abbreviations: cerebral amyloid angiopathy, CAA; interquartile range, ICR;

*according to modified Boston criteria

Supplementary Figure 1

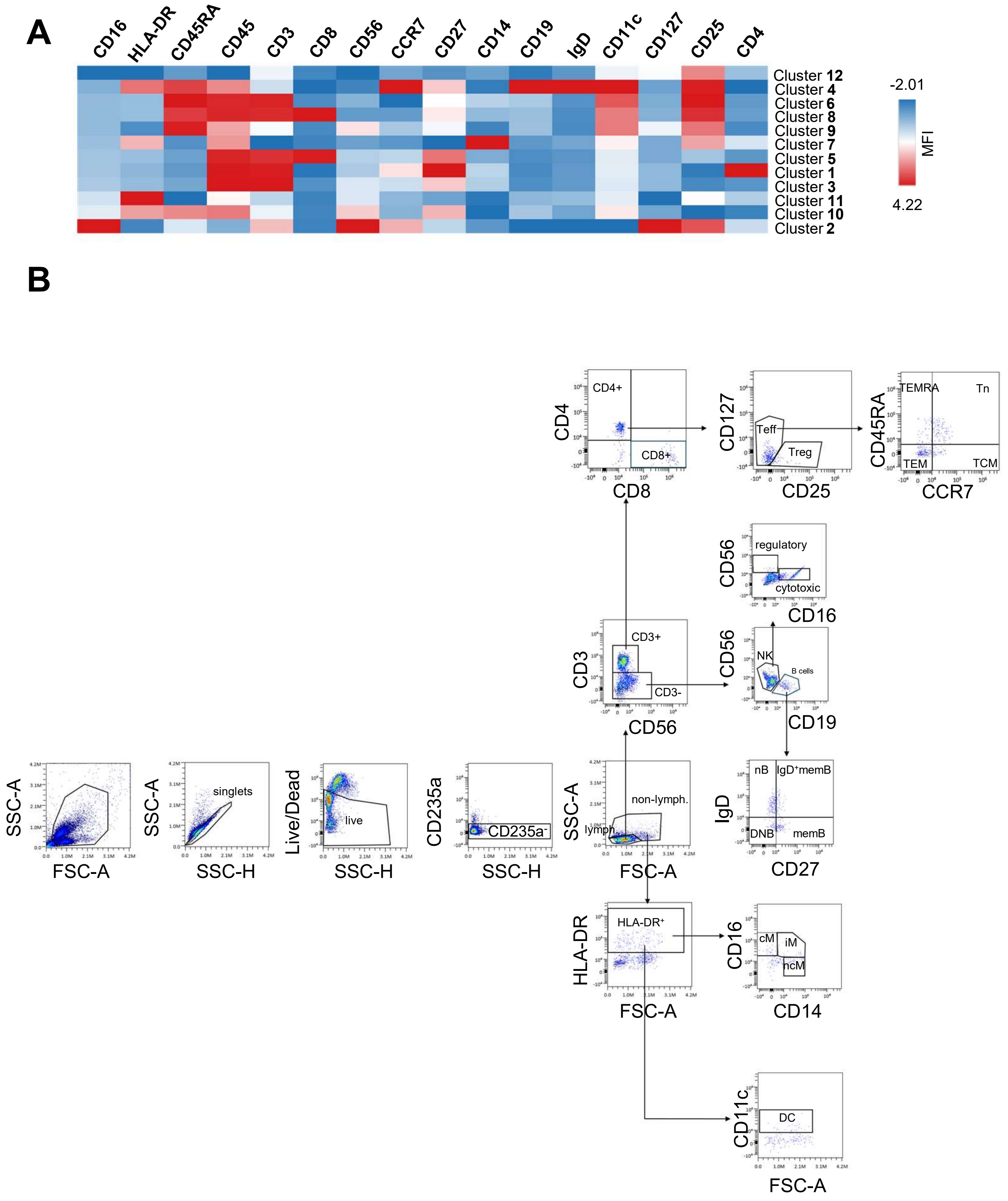

Supplementary Figure 2

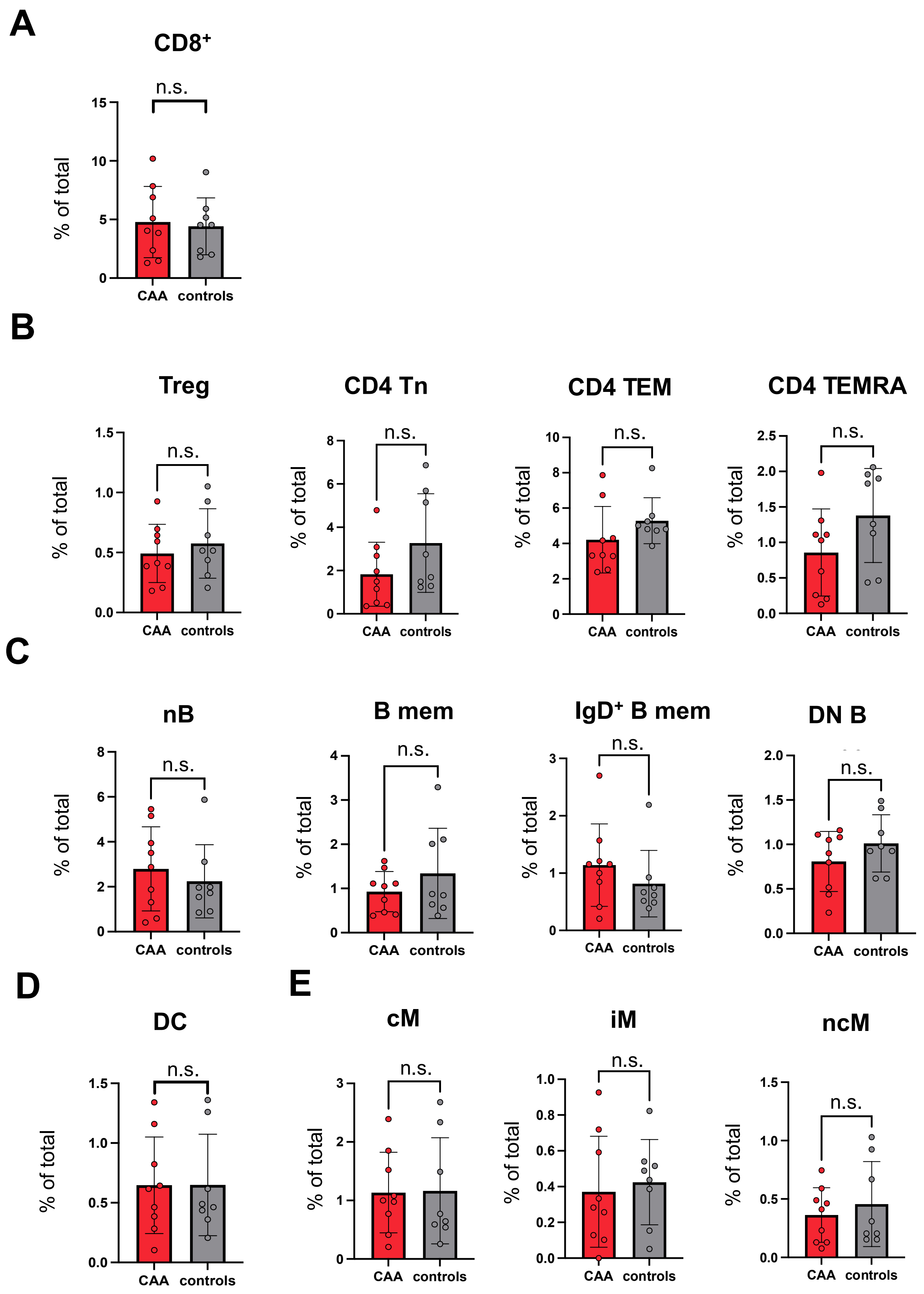

Figure legends

Supplementary Figure 1. Characterization of immune cell populations using FlowSOM clustering and detailed flow cytometry gating strategy

(A) Marker expression profiles of immune cell clusters identified by FlowSOM

Median fluorescent intensities (MFIs) of marker expression on clusters identified by FlowSOM clustering (k = 12).

(B) Gating strategy for high-dimensional flow cytometry analysis of immune cell subsets.

Representative gating strategy for high-parameter flow cytometry surface staining of cells from whole blood samples. Non-lymphocytes (non-lymph.) include dendritic cells, monocytes, and other non-lymphoid cells, while lymphocytes (lymph.) include T cells, B cells, and natural killer (NK) cells. Monocyte subsets were defined as classical monocytes (cM; $CD14^{+}CD16^{-}$), intermediate monocytes (iM; $CD14^{+}CD16^{+}$), and non-classical monocytes (ncM; $CD14^{-}CD16^{+}$). Dendritic cells (DCs) were identified as HLA-$DR^{+}CD11c^{+}$ cells. NK cell subsets were categorized into regulatory NK cells ($CD56^{bright}CD16^{-}$) and cytotoxic NK cells ($CD56^{dim}CD16^{+}$). T cell subsets included T effector cells (Teff), naïve $CD4^{+}$ T cells (Tn; $CD45RA^{+}CCR7^{+}$), regulatory T cells (Treg; $CD3^{+}CD4^{+}CD25^{+}CD127^{-}$), effector memory $CD4^{+}$ T cells (TEM; $CCR7^{-}CD45RA^{-}$), effector memory $CD4^{+}$ T cells re-expressing CD45RA (TEMRA; $CCR7^{-}CD45RA^{+}$), and central memory $CD4^{+}$ T cells (TCM; $CCR7^{+}CD45RA^{-}$). B cell subsets included naïve B cells (nB; $CD19^{+}IgD^{+}CD27^{-}$), memory B cells (mem B; $CD19^{+}CD27^{+}IgD^{-}$), IgD^{+} memory B cells (IgD^{+} mem B; $CD19^{+}CD27^{+}IgD^{+}$), and double-negative B cells (DN B; $CD19^{+}CD27^{-}IgD^{-}$).

Supplementary Figure 2. Comparison of immune cell subset abundance in CAA patients vs. control group: Frequencies of major immune populations

Frequencies (% of total leukocytes) of selected immune cell populations in peripheral blood of patients with cerebral amyloid angiopathy (CAA) compared to controls. Statistical analysis: unpaired two-tailed t-test with Welch's correction; * $p < 0.05$. "ns" = not significant.

(A) $CD8^+$ T cell frequencies

Comparison of cytotoxic $CD8^+$ T cells between CAA patients and controls.

(B) $CD4^+$ T cell subset distribution

Frequencies of regulatory T cells (Treg: $CD3^+CD4^+CD25^+CD127^-CD56^-CD8^-$), naïve $CD4^+$ T cells (Tn: $CD45RA^+CCR7^+$), effector memory (TEM: $CCR7^-CD45RA^-$), and terminally differentiated effector memory cells re-expressing CD45RA (TEMRA: $CD45RA^+CCR7^-$).

(C) B cell subset composition

Comparison of naïve B cells (nB: IgD^+CD27^-), memory B cells (mem B: $CD27^+IgD^-$), IgD^+ memory B cells (IgD^+ mem B: $CD27^+IgD^+$), and double-negative B cells (DN B: $CD27^-IgD^-$).

(D) Dendritic cell frequencies

Frequencies of conventional dendritic cells (DCs: $HLA\text{-}DR^+CD11c^+$) in CAA versus control samples.

(E) Monocyte subset distribution

Frequencies of classical (cM: $CD14^+CD16^-$), intermediate (iM: $CD14^+CD16^+$), and non-classical monocytes (ncM: $CD14^-CD16^+$).
